# The Source of Power Matters: Positional Power as a Better Predictor of Sexual Interest Perceptions than Dispositional Power Among Men within a Military Context

**DOI:** 10.1007/s10508-021-02255-7

**Published:** 2022-03-07

**Authors:** Pei Hwa Goh, Peter Lucas Stoeckli, Dominik Schoebi, Hubert Annen

**Affiliations:** 1 Department of Psychology, Monash University—Malaysia Campus, Bandar Sunway, Subang Jaya, Selangor Malaysia; 2 Department of Military Psychology and Pedagogy, Military Academy at ETH Zurich, 8903 Birmensdorf, Switzerland; 3 Department of Psychology, University of Fribourg, Fribourg, Switzerland

**Keywords:** Perception of sexual interest, Power, Mating, Military

## Abstract

The current research examined the roles of positional power induced by one’s hierarchical position in an organization and dispositional power (i.e., one’s general feeling of power) in the perception of sexual interest in a military context. In two vignette-based experiments with men who were military members, positional power induced by military rank led to heightened sexual perceptions. Men estimated higher sexual interest from their interaction partner when interacting with a hypothetical woman of a lower military rank, compared to a woman of equal (Experiment 1; *N* = 144) or higher military rank (Experiment 2; *N* = 232). Being in a relatively higher rank induces feelings of power over the interaction partner and thus results in a higher perception of sexual interest. Furthermore, Experiment 2 revealed that positional power better predicted heightened perceived sexual interest than dispositional power.

## Introduction

Is this person sexually interested in me or not? Finding the correct answer to this question is crucial, given the possibility that misperceiving sexual interest could result in unwanted sexual *mis*behaviors such as sexual harassment (e.g., Gruenfeld et al., 2008). Despite the importance of correctly perceiving sexual interest, this accuracy is difficult to achieve as sexual interest is often conveyed in an indirect, ambiguous manner. Sexual interest communication involves the use of nonverbal cues like smiling, physical touch, interpersonal proximity, and eye contact (e.g., Abbey & Melby, 1986; Muehlenhard et al., 1986), which also convey general interest and friendliness. Thus, individuals typically rely on other sources of information in addition to the actual behaviors directed toward them when judging the sexual interest of others. Some of this information is derived from the interaction partner or target. For instance, men perceive higher sexual interest from women who are more attractive (Treat et al., 2016), dress in red rather than green, white, or blue (Guéguen, 2012; Pazda et al., 2012), or wear clothing that reveals more skin (Guéguen, 2011). Another source of information stems from the perceiver. For instance, perception of sexual interest has been shown to vary according to the perceiver’s mood states and cultural values (Goh et al., 2018).

Another robust perceiver-based driver of sexual perception is an individual’s power. Relative to powerless individuals, those in power are more likely to think that another person is sexually interested in them (Kunstman & Maner, 2011). Even though previous research has already established the link between power and sexual perception (Gruenfeld et al., 2008; Kunstman & Maner, 2011), further examination is warranted. Despite evidence suggesting that perpetrators in 67% of sexual harassment cases in the U.S. military were military personnel of a higher rank (Morral et al., 2015), which suggest that people in power may particularly be prone to sexual misperception in workplace settings, existing findings (Kunstman & Maner, 2011) have been derived from university student samples who underwent social power manipulations. These findings may not directly apply to organizational contexts or other aspects of power, given evidence suggesting contingencies of power effects (Galinsky et al., 2015). In light of psychology’s replication crisis (e.g., Open Science Collaboration, 2015), we sought to replicate Kunstman and Maner’s findings within an organizational setting. We added a new direction to the existing research by considering the roles of both positional power, which refers to a sense of power that having a higher rank or position in an organization affords, and one’s personal sense of power, which is relatively stable across situations and relationships (Anderson et al., 2012). Hence, the current study aimed to shed light on the question of whether one’s personal sense of power or power from having a higher rank at an organization better predicts sexual interest perception, which may have important implications for potential measures against sexual harassment.

### Power and Sexual Perception

Power can be defined in terms of one’s control over resources, through which influence over others can be achieved (Fiske, 1993). People in power possess more control over valuable assets, which can be used to achieve their goals and grant relative immunity to interpersonal punishments. According to the approach-inhibition theory of power (for a review, see Keltner et al., 2003), being in power activates a tendency to approach rewards (e.g., attention to rewards, automatic cognition, disinhibited behavior) while a lack of power activates inhibitory tendencies (e.g., attention to threats, systematic cognition, inhibited behavior). Power’s activation of the approach system may extend to the activation of goals related to sex and mating since this approach system regulates functions that are fundamental to survival (Depue & Collins, 1999; Gray, 1973). This includes sex, which is a primary reward that is essential for reproduction and gene propagation (Hull, 1943). Indeed, power has been found to automatically activate sexual concepts (Bargh et al., 1995) and prime mating goals (Kunstman & Maner, 2011), which may in turn sexualize interpersonal perceptions. In line with this, Kunstman and Maner found that participants in the power group, who were led to believe that they were the most qualified to lead their team, expected more sexual interest from their subordinate team members than participants in the control group.

Past research has induced power via role assignment following false feedback of high leadership abilities (Gruenfeld et al., 2008; Kunstman & Maner, 2011) or via experiential priming, where participants are asked to recall a personal situation in which they had power over another person (Galinsky et al., 2003). In the present research, however, power manipulation was achieved through the hierarchical position or the rank contrast between the perceiver and interaction partner without the provision of false feedback of subjective social power. We hypothesized that participants who hold ranks that are higher than their interaction partner (high positional power) would perceive higher sexual interest than participants who hold a relatively lower rank (low positional power).

### Dispositional Power as a Moderator

Extant research has highlighted the importance of the role of one’s dispositional level of power or one’s general sense of power across situations and relationships (Anderson et al., 2012) as a moderator of the positional power effect (Galinsky et al., 2015). However, these findings have been mixed. One line of findings suggests that positional power effects are limited to individuals who lack power by disposition (Bugental & Lewis, 1999; Williams et al., 2017). For instance, Williams and colleagues found that only individuals with chronically low levels of power demonstrated heightened hostility, sexual harassment tendencies, and harassing behaviors in response to experimentally induced power. Second, dispositional power has been shown to amplify the effect of power manipulations, in that individuals with more dominant personalities show more susceptibility to power manipulations (Anderson & Berdahl, 2002). In the current work (Experiment 2), we examined whether the effect of positional power on sexual perception is qualified or amplified by one’s dispositional sense of power in a military context.

### The Present Research

Given important implications of the power and sexual perception link within organizations with cultures that place a high value on hierarchy (Zaleski, 2015; Zurbriggen, 2010), we conducted two vignette-based experiments on male military members of the Swiss Armed Forces. In Experiment 1, we examined whether positional power, induced by the military rank of the perceiver, increased sexual interest perception. In Experiment 2, we manipulated power via the variation of the interaction partner’s rank and additionally examined whether the positional power effect is robust across levels of dispositional power.

### Experiment 1

The aim of the first experiment was to examine the effect of positional power on the perception of sexual interest. We hypothesized that military members who held positional power would perceive higher sexual interest from an interaction partner of the other sex compared to those who lack positional power.

## Method

### Participants

A total of 157 men who were officer candidates of the Swiss Armed Forces completed a short questionnaire as part of fulfilling the Joint Officer Training Course (JOTC) requirements. The JOTC, which is run three times a year, is a selective admission course that teaches candidates the basic skills required of an officer. Admission into this course is limited to military members who have demonstrated leadership potential during their basic training, and good leadership abilities as a leader of a squad, which consists of five to ten recruits, at cadre school. Our sample consisted of officer candidates with three months to two years of military experience, who mostly held the rank of private first class. Participants were randomly allocated into the power or control conditions. After removing participants who failed the attention check and those with incomplete responses in the perception of sexual interest measure, we were left with a total of 144 participants aged 18 – 29 years (*M* = 20.7, *SD* = 1.7). Of these, 69 were in the power condition while 75 were in the control condition.

### Measures and Procedure

Prior to participation, participants were first informed that the study aimed to examine how women in the military are perceived by their male counterparts. The researchers also informed them that the participation is completely anonymous and voluntary. Participants were then given a written hypothetical scenario, which portrayed a casual encounter in a military setting with a woman who is a recruit, as shown in the Appendix. The woman was described as (1) attractive, as this has been shown to activate mating goals (Maner et al., 2005) and (2) approaching the participant, as contact initiation is viewed as a nonverbal indicator of general interest (Koeppel et al., 1993). To manipulate positional power, participants were asked to imagine themselves as a staff sergeant (power condition) or as a recruit (control condition).

To check if participants understood the situation correctly or read it carefully, we included an attention check question, i.e., “The difference in ranks was clearly stated,” which was rated on a 7-point Likert scale (1 = *not at all*, 7 = *most definitely*). The vignettes were designed to show a clear hierarchical difference in the power condition, but no difference in the control condition. Participants who rated three and below in the power condition or three and above in the control condition were excluded from our analyses.

To examine whether the positional power manipulation was successful, participants provided their momentary feelings of power by rating the item “I have power over the target.” Two items, “I think that she would be interested in a sexual encounter with me” and “I think that she was flirting with me” (*r* = 0.60), were used to capture the perception of sexual interest. All items were rated on 7-point Likert scales (1 = *not at all,* 7 = *most definitely*).

## Results and Discussion

### Positional Power Manipulation Check

Participants who imagined themselves to be a staff sergeant reported experiencing higher momentary power over the interaction partner (*M* = 4.6, *SD* = 1.8) than those who imagined to be a recruit (*M* = 2.8, *SD* = 2.0), *t*(141.93) = 5.72, *p* < 0.001, *d* = 0.95, 95% CI [0.60, 1.29]. The significant difference in momentary power between the power and control conditions suggests that the positional power manipulation using rank differences was successful.

### The Effect of Positional Power on Perception of Sexual Interest

Participants in the power condition reported higher sexual interest from the interaction partner (*M* = 4.1, *SD* = 1.6) than those in the control condition (*M* = 3.6, *SD* = 1.4), *t*(142) = -2.09, *p* = 0.038, *d* = 0.35, 95% CI [0.02, 0.68]. This supports our hypothesis and is in line with the findings of Kunstman and Maner (2011).

### Momentary Feelings of Power as Mediator

To further examine whether or not momentary feelings of power mediated the effects of positional power on sexual interest perception, regression analysis following the framework outlined by Hayes (2013; SPSS macro: Process Model 4) was conducted. Results indicated that positional power was a significant predictor of subjective feelings of power, *B* = 1.80, 95% CI [1.17, 2.42], and perceived sexual interest, *B* = 0.52, 95% CI [0.03, 1.02]. Mediation analyses based on 10 000 bootstrapped samples using bias-corrected and accelerated 95% confidence intervals showed that positional power had a nonsignificant residual direct effect, *DE* = 0.20, 95% CI [-0.34, 0.73], and a significant indirect effect via subjective feelings of power, *IE* = 0.32, 95% CI [0.11, 0.62]. These results suggest that subjective feelings of power completely mediated the relationship between positional power and perception of sexual interest – providing further support for the utility of relative rank in inducing positional power.

### Experiment 2

The first experiment provided evidence that holding positional power increases male military members’ estimation of a woman’s sexual interest by increasing subjective, momentary feelings of power over the interaction partner. However, manipulating power by asking participants to imagine holding a specific military rank could have potentially influenced our results. Participants had to adopt a position that either they have yet to experience (staff sergeant in the power condition) or requires retrospective recall (recruit in the control condition) – both of which could be susceptible to biases, making it potentially challenging for them to respond from their own perspective (Swartzman & McDermid, 1993). To account for these potential biases, we induced power by manipulating the rank of the interaction partner in Experiment 2 to replicate the results of Experiment 1. We expected participants with positional power to perceive higher sexual interest (Hypothesis 1) than those lacking in positional power.

In addition, Experiment 2 also investigated whether the effect of positional power is conditional on individual differences in dispositional power, which was measured using the Personal Sense of Power Scale (Anderson et al., 2012). Because this scale had not been validated in a Swiss sample at the time of the study, we additionally measured participants’ self-esteem to test if our sample demonstrates the positive association between self-esteem and the personal sense of power as found in Anderson et al.’s study.

Given the mixed findings in the literature, we proposed two variations of our moderation hypothesis. First, the positive effect of positional power on the perception of sexual interest would only be present among those who are lower in power by disposition (Hypothesis 2a; Bugental & Lewis, 1999; Williams et al., 2017). Following Anderson and Berdahl (2002), however, the positional power effect would only be present among those who are higher in power by disposition (Hypothesis 2b).

## Method

### Participants

A total of 233 men, who were officer candidates of the Swiss Armed Forces participated in this experiment as partial fulfillment of the JOTC requirements. They were randomly allocated into the high and low power conditions. Twelve participants were excluded because they failed the manipulation check. Another participant was excluded because he did not complete the dispositional power measure and demographics, leaving a final sample of 232 (218 privates first class, 10 sergeants, 2 staff sergeants, 2 unknown) with a mean age of 20.6 years (*SD* = 2.1).

### Measures and Procedure

Participants read a written scenario, which described an encounter with an attractive woman, who was a member of the Swiss Armed Forces, in a military setting. Positional power was manipulated by varying the woman’s rank (private vs. lieutenant; the rank of a private is lower than the rank of a sergeant or staff sergeant while the rank of a sergeant or staff sergeant is lower than the rank of a lieutenant). Participants completed the same set of items from Experiment 1 to measure their perceptions of sexual interest and subjective feelings of power over the interaction partner. All items were rated on 7-point Likert scales (1 = *not at all*, 7 = *most definitely*).

Participants then completed the Personal Sense of Power Scale (Anderson et al., 2012), which is an eight-item measure of dispositional power (e.g., “I can get people to do what I want”). Participants were asked to rate how much they agreed to these statements on a scale of 1 (*disagree strongly*) to 7 (*agree strongly)* with regard to their interpersonal relationships in general, α = 0.77.

Self-esteem was measured using Rosenberg’s Self-Esteem Scale (Rosenberg, 1965; Roth et al., 2008). Participants provided ratings to ten items (e.g., “all in all, I take a positive attitude toward myself”) on a scale of 1 (*not at all*) to 5 (*very),* α = 0.80. Item scores were averaged to form a self-esteem score, where higher scores indicated higher self-esteem.

## Results and Discussion

### The Validation of the Personal Sense of Power Scale

A principal components analysis was conducted to examine the factor structure of the dispositional power measure. Two factors (with eigenvalues exceeding 1) were identified as underlying the eight-item questionnaire. The first factor, which comprised of the four negatively-worded items (i.e., “My wishes do not carry much weight,” “Even if I voice them, my views have little sway,” “My ideas and opinions are often ignored,” and “Even when I try I am not able to get my way”), accounted for 40.47% of the variance and appeared to tap into a sense of social powerlessness, α = 0.73. The second factor, which comprised of the four positively-worded items (i.e., “I can get people to listen to what I say,” “I can get people to do what I want” “I think I have a great deal of power,” “If I want, I get to make the decisions”) accounted for only 14.28% of the variance in the data and were more reflective of a sense of power or influence, α = 0.63. Given the poor loadings and internal consistency of the second factor, we examined dispositional sense of power using responses to the item, “I think I have a great deal of power,” as single-item measures of power have been shown to reliably and validly capture individual differences in power (e.g., Lammers & Stoker, 2019; Lammers et al., 2011). Zero-order correlations between these measures and others used in this study are presented in Table 1.

**Table 1:** Intercorrelations and descriptive statistics for power variables, self-esteem, perception of sexual interest, and age

	1	2	3	4	5	6	7	8
1. Dispositional power: Composite								
2.Dispositional power: Single-item power measure	.47***							
3. Dispositional power: Sense of powerlessness	− .88***	− .18**						
4. Momentary feelings of power	− .01	.25***	.06					
5. Global self-esteem	.52***	.14*	− .53***	− .14*				
6. Perception of sexual interest	.05	.13*	.01	.29***	.00			
7. Age	.05	.09	− 03	.03	− 16*	− .03		
8. Positional power (manipulated)	− .06	.03	.09	.22**	.04	.23***	.02	
*M*	5.5	3.9	2.3	2.6	4.3	3.3	20.6	–
*SD*	0.7	1.3	0.8	1.7	0.5	1.5	2.1	–
*N*	232							

^*^p < .05, **p < .01, ***p < .001

Positional power was dummy-coded as 0 = lower power relative to target and 1 = higher power relative to target. The degree of freedom for all correlations was 230

To further test the validity of the dispositional power measure(s), we examined their associations with global self-esteem. In line with Anderson and colleagues (2012; Study 5) who found a positive correlation between power and self-esteem (*r* = 0.45, *p* < 0.01), we found in our sample that the single-item sense of power measure, the four-item sense of powerlessness subscale, and the composite personal sense of power measure were significantly correlated with global self-esteem.

### Positional Power Manipulation Check

*t*-tests were conducted to examine the effect of positional power manipulation on feelings of power. As shown in Table 2, participants reported feeling more in power when imagining a woman holding a lower rank than a woman holding a higher rank relative to themselves, *d* = 0.47, 95% CI [0.73, 0.21]. Given that dispositional power was measured after the manipulation, we also tested whether the manipulation affected participants’ ratings of dispositional power. Ratings on the sense of powerlessness and sense of power, which includes both the composite score and single-item measure, did not vary by positional power. All in all, these findings suggest that the positional power was successful in inducing momentary feelings of power, and that dispositional power was not affected by the manipulation.

**Table 2:** *t*-test results comparing momentary and dispositional power measures by rank condition

	Low positional power		High positional power		*t*-test
	*M*	SD	*M*	SD	
Dispositional power: Composite	5.5	0.6	5.4	0.7	0.79
Dispositional power: Single-item power measure	3.8	1.2	3.9	1.3	− 0.85
Dispositional power: Sense of powerlessness	2.2	0.7	2.4	0.9	−1.38
Momentary feelings of power	2.2	1.3	2.9	1.9	-3.57***
*N*	114		118		

***p < .001. Scores ranged from 3.38 to 7.00 for composite scores of dispositional power; 1.0–7.0 for single-item power measure; 1.00–5.25 for sense of powerlessness; and 1.00–7.00 for momentary feelings of power. The degree of freedom for all t-tests were 230 except for momentary feelings of power, which violated the assumptions for homogeneity of variance and resulted in the corrected degree of freedom of 207.06

Of the dispositional power measures, only the single-item measure of the overall sense of power was shown to have a significant positive correlation with momentary feelings of power (see Table 1). A general sense of powerlessness and the composite personal sense of power score did not correlate significantly with momentary feelings of power.

To examine if the effects of positional power were conditional on individual differences in the overall sense of power and powerlessness, we conducted a regression analysis following the framework outlined by Hayes (2013; SPSS macro: Process Model 2). As shown in Table 3, dispositional power did not moderate the effect of positional power on momentary feelings of power. Only positional power and overall sense of power emerged as significant predictors of momentary feelings of power. We ran the same model without the interaction terms and found that the pattern of findings remains unchanged. Participants who felt that they generally possessed a great deal of power in their social relationships (β = 0.26) and those who imagined interacting with a woman of a lower military rank (β = 0.20) reported higher momentary feelings of power over the interaction partner compared to those who felt less in power in daily life and those who imagined interacting with a woman of a higher military rank. In sum, these results suggest that the manipulation of positional power appeared to be consistently effective across varying levels of dispositional power. Also, personal sense of power was a relatively better predictor of momentary feelings of power over the interaction partner than positional power.

**Table 3:** Moderation model coefficients for positional power predicting momentary feelings of power and perception of sexual interest conditional on dispositional power measures, i.e., sense of power and sense of powerlessness

Variable	Momentary feelings of power		Perception of sexual interest	
	*B*	95% CI	*B*	95% CI
Constant	2.47	1.13, 5.07	4.24**	2.03, 6.46
Sense of power	0.33***	0.15, 0.51	0.16	− 0.02, 0.34
Positional power	0.66***	0.25, 1.07	0.69**	0.30, 1.08
Positional power x Sense of power	0.32	− 0.05, 0.68	0.08	− 0.28, 0.45
Sense of powerlessness	0.20	− 0.06, 0.47	0.02	− 0.23, 0.28
Positional power x Sense of powerlessness	− 0.04	− 0.57, 0.50	-0.02	− 0.53, 0.48
Age	0.00	− 0.12, 0.13	-0.05	− 0.15, 0.06

^*^p < .05, **p < .01, ***p < .001

Positional power was dummy-coded as 0 = lower power relative to target and 1 = higher power relative to target. Four participants were removed as they were identified as multivariate outliers, leaving a final sample size of 228 (*M*_age_ = 20.41, *SD*_*age*_ = 1.52) for our analyses

### Perception of Sexual Interest

The same moderation analysis was conducted with the perception of sexual interest as the outcome variable. As shown in Table 3, only positional power emerged as a significant predictor. Overall sense of power and powerlessness neither predicted nor interacted with positional power to predict the perception of sexual interest – rejecting Hypotheses 2a and 2b. We then re-ran the model excluding the interaction terms and found that sense of power (*B* = 0.16, *p* = 0.046) also significantly predicted perception of sexual interest in addition to positional power (*B* = 0.69, *p* = 0.001). Sense of powerlessness and age did not significantly predict the perception of sexual interest, *p*s > 0.250. The standardized coefficients suggest that positional power (β = 0.23) remains superior to dispositional power (β = 0.13) when predicting sexual interest perceptions. This was confirmed with additional analyses, which showed that the addition of dispositional power indicators as predictors into the model did not significantly improve the model fit from the model with positional power and age as predictors, Δ*R*^*2*^ = 0.07, *F*(1, 223) = 2.03, *p* = 0.134.

## General Discussion

The current study was the first to examine the roles of various forms of power in predicting the perception of sexual interest among men within an organizational context. Across two experiments, we found consistent evidence for the effect of positional power on men’s sexual perceptions within a military context. Men estimated higher levels of sexual interest from a hypothetical female colleague when they held a military rank that was higher, rather than equal to or lower, than the female colleague. Our results support the findings of Kunstman and Maner (2011), are in line with other previous findings on power’s association with sexual cognitive concepts (Bargh et al., 1995) as well as the approach-inhibition theory of power (Keltner et al., 2003).

Second, our findings suggest that, at least in the context of the military, the effect of power on sexual interest perception stems more from one’s rank than one’s dispositional sense of power. More importantly, the effect of positional power on the perception of sexual interest does not appear to be contingent on one’s overall sense of power or powerlessness. In line with this, there is some evidence that situational rather than personality-based cues tend to be more important in workplaces since they convey information regarding behavioral expectations and outcomes (James et al., 1990; Scott & Bruce, 1994). Moreover, power is, by virtue, a relational concept that is context-dependent (Emerson, 1962; Thibaut & Kelley, 1959). It is therefore justifiable that one’s power in the situation would contribute more to their sexual perception than their dispositional sense of power in a professional, military setting.

All in all, the current work highlights the importance of taking into account multiple sources of power when examining power effects. Even though one’s position in the organization played a larger role in predicting perception of sexual interest than dispositional power, we found that moment-to-moment feelings of power over a colleague were informed by not only one’s hierarchy in the workplace but more so by one’s general feelings of social power. That one’s momentary feeling of positional power is driven both by one’s rank and personal sense of power is not surprising. However, the inconsistent results on the superiority of positional power as a predictor and the absence of a test of the mechanism by which positional power shapes the perception of sexual interest call for a need to examine other power-related mediators of this effect.

For instance, future studies could examine a more subjective evaluation of momentary sense of power, which is less specific to the target or interaction partner, in addition to subjective feelings of positional power, which is specific to the interaction partner. Anderson and Berdahl (2002; Study 2) demonstrated the function of momentary subjective feelings of power as a mediator of the effect of positional power on perceptions of a partner’s liking. Participants assigned to high power conditions, relative to low power conditions, felt more in power and thus perceived more liking from their interaction partners. However, subjective feelings of power may not merely represent a path by which positional power predicts perceptions. Instead, these two forms of power can also independently predict perceptions, and in some cases, even interact to predict certain outcomes such as one’s mood state (Smith & Hofmann, 2016). In the current work, we only measured the target-specific momentary feeling of power over a colleague rather than a subjective sense of power at that moment. A more nuanced understanding of the different momentary power effects would better inform the development of interventions to improve workplace communication and reduce sexual harassment.

The interpretation of current findings may be limited due to the sole reliance on vignettes to test our hypotheses. While vignette responses have been found to translate into reactions in real situations and can produce valuable results (Aguinis & Bradley, 2014; De Cremer & van Knippenberg, 2004), future studies should consider incorporating behavioral observations during face-to-face interaction tasks or implicit measures of sexual interest to address this limitation, and further complement our findings. Studies involving dyadic exchanges would also allow us to test for actor and partner effects. That is, we could examine whether the effect of power would increase the perceiver’s expectations of sexual interest (actor effects) or that the perceiver’s power would also increase the sexual interest and behaviors of the interaction partner, which would, in turn, increase the perceiver’s estimations of sexual interest (partner effects; Eastwick et al., 2013). Nevertheless, our findings largely are in line with previous work, which has employed laboratory-based interactions (Anderson & Berdahl, 2002; Kunstman & Maner, 2011) and more naturalistic experience-sampling methods (Smith & Hofmann, 2016).

It would also be worthwhile to replicate the current study in organizations with a more gender-balanced distribution of members. This would allow researchers to test whether current findings extend to women, or if there are reliable gender differences in the way that various forms of power predict the perception of sexual interest. Based on past findings (Kunstman & Maner, 2011; Lammers & Stoker, 2019), we do not expect current findings to differ by the gender or sex of the perceiver. Furthermore, researchers could also examine whether or not the cardinal role of positional power in sexual perception demonstrated in the current study is specific to organizations with a strong cultural endorsement of power hierarchies such as the military (e.g., Zaleski, 2015).

Overall, the current research offers useful insights, which could be applied in preventive interventions or training programs that target sexual harassment in military contexts. Our findings suggest that it could be worthwhile for such programs to concentrate not only on the definition of harassing behaviors and the post-harassment reporting process but also on the understanding of possible psychological “side-effects,” such as perceptual biases, that may come automatically with a higher rank.

## Data Availability

The datasets used in the current study are available from the corresponding author on reasonable request.

## Appendix

Scenarios for the Power and Control conditions in Experiment 1.

You are a *staff sergeant* [Control: *recruit*] in the 6th week of the basic military training course, and you are about to enter the dining area for dinner. On your way there, you meet recruit Stefanie Rocheray, whom you have never met. Recruit Stefanie Rocheray is attractive and smiles at you before approaching you to ask about the “optional off-base evening” tomorrow evening. Specifically, she asks for a restaurant recommendation near the barracks. You respond with a witty remark. She laughs and responds with another funny remark. You eventually recommend her a good pizzeria, for which she thanks you.
